# Dyssynchronous Left Ventricular Activation is Insufficient for the Breakdown of Wringing Rotation

**DOI:** 10.3389/fphys.2022.838038

**Published:** 2022-05-09

**Authors:** Tobias Gerach, Stephanie Appel, Jacek Wilczek, Krzysztof S. Golba, Tomasz Jadczyk, Axel Loewe

**Affiliations:** ^1^ Institute of Biomedical Engineering, Karlsruhe Institute of Technology (KIT), Karlsruhe, Germany; ^2^ Department of Electrocardiology, Upper-Silesian Heart Center, Katowice, Poland; ^3^ Department of Electrocardiology and Heart Failure, Medical University of Silesia, Katowice, Poland; ^4^ Division of Cardiology and Structural Heart Diseases, Medical University of Silesia, Katowice, Poland; ^5^ Interventional Cardiac Electrophysiology Group, International Clinical Research Center, St. Anne’s University Hospital, Brno, Czech Republic

**Keywords:** cardiac mechanics, finite element simulation, electromechanical mapping, wringing, torsion, Lagrangian particle tracking, NOGA XP

## Abstract

Cardiac resynchronization therapy is a valuable tool to restore left ventricular function in patients experiencing dyssynchronous ventricular activation. However, the non-responder rate is still as high as 40%. Recent studies suggest that left ventricular torsion or specifically the lack thereof might be a good predictor for the response of cardiac resynchronization therapy. Since left ventricular torsion is governed by the muscle fiber orientation and the heterogeneous electromechanical activation of the myocardium, understanding the relation between these components and the ability to measure them is vital. To analyze if locally altered electromechanical activation in heart failure patients affects left ventricular torsion, we conducted a simulation study on 27 personalized left ventricular models. Electroanatomical maps and late gadolinium enhanced magnetic resonance imaging data informed our in-silico model cohort. The angle of rotation was evaluated in every material point of the model and averaged values were used to classify the rotation as clockwise or counterclockwise in each segment and sector of the left ventricle. 88% of the patient models (*n* = 24) were classified as a wringing rotation and 12% (*n* = 3) as a rigid-body-type rotation. Comparison to classification based on *in vivo* rotational NOGA XP maps showed no correlation. Thus, isolated changes of the electromechanical activation sequence in the left ventricle are not sufficient to reproduce the rotation pattern changes observed *in vivo* and suggest that further patho-mechanisms are involved.

## 1 Introduction

In the healthy human heart, left ventricular (LV) ejection and filling is supported by the twisting and untwisting of the ventricle during systole and diastole, respectively. This twisting or wringing motion is determined by several anatomical and physiological features such as age, contractility, structure of the myocardium, and muscle fiber orientation (Omar et al., 2015). Furthermore, the electrical activation pattern of the LV is heterogeneous due to the His-Purkinje system and the anisotropic conduction of the electrical potential (Sengupta et al., 2007). Consequently, the activation pattern of the LV follows an endocardial to epicardial direction. Combined with the counter-directional helical arrangement of the endo- and epicardial muscle fibers, this results in a clockwise and counterclockwise rotation of the basal and apical segments, respectively.

In pathological cases, this wringing motion of the LV can be disrupted by dyssynchronous mechancial activation resulting in a reduced LV ejection fraction (EF) (Rüssel and Götte, 2011). Dyssynchrony may originate from different sources such as an abnormal electrical activation in patients with left bundle branch block (LBBB) or post-ischemic remodeling and geometric alterations in heart failure patients (Sillanmäki et al., 2018; Paoletti Perini et al., 2016). Multiple studies confirmed changes in LV rotational behavior in heart failure patients using MRI tagging and speckle tracking echocardiography (Popescu et al., 2009; van Dalen et al., 2008a; Sade et al., 2008; Rüssel et al., 2009a). Setser et al. (2003) specifically observed rigid-body type (RBT) rotation in patients with end-stage heart failure, meaning apical and basal segments were rotating in the same direction. Cardiac resynchronization therapy (CRT) with an implanted device is often used in patients showing ventricular dyssynchrony in an attempt to restore LV EF. However, around 30–40% of patients do not respond to this kind of intervention (Daubert et al., 2012). One reason might be a bad choice for the pacing site (Leclercq et al., 2019). Therefore, it is important to optimize CRT parameters for each patient and LV rotation has become increasingly important for this purpose (Rüssel et al., 2009b).

Jadczyk et al. (2021) investigated electromechanical coupling and scar tissue burden with respect to rotational patterns observed in patients showing heart failure with reduced ejection fraction (HFrEF) and LBBB. In their cohort of 30 patients, they found six cases showing normal wringing rotation and 24 cases showing RBT rotation. They concluded that remodeling changes the physiological gradient in electromechanical activation, which causes regional delays in mechanical activation and thus dyssynchronous contraction of the LV. In contrast, following a physiological propagation of electrical and mechanical activation, an intact electromechanical coupling (with constant electromechanical delay) will result in a wringing motion. However, due to the small number of study participants the results by Jadczyk et al. (2021) should be considered with caution. To elucidate the role of the different contributing mechanisms suggested by Jadczyk et al. (2021), we performed an in silico study under controlled conditions informed by their *in vivo* electromechanical mapping data. Specifically, we hypothesized that the altered electrical activation pattern is sufficient to change wringing rotation to RBT. Spatiotemporal electromechanical parameters including local activation time (LAT), local rotational electromechanical delay (LEMD), and total rotational electro-mechanical delay (TEMD) were combined with local scar burden derived from late-gadolinium-enhanced cardiac magnetic resonance imaging (LGE-MRI) and incorporated into the LV model. LV rotational patterns are analyzed and classified into two groups defined as normal wringing rotation and RBT. Finally, the classification based on the simulation results is compared to the clinical classification.

## 2 Materials and Methods

### 2.1 Anatomical Finite Element Model

As a representation of the LV, we used a truncated ellipsoid with varying wall thickness. The wall thickness changes from 7 mm at the base to 3.5 mm at the apex. With a sphericity index of 1.58, the ellipsoid has a similar shape as the left ventricles of the patient cohort in Jadczyk et al. (2021). The meshes were created in Gmsh (Geuzaine and Remacle, 2009) using a fully parameterized workflow. Spatial discretization was done using the finite element method with a total of 9,237 quadratic tetrahedral elements (P2) for the LV with 53,019 degrees of freedom. Based on spatial convergence results presented in a previous study (Gerach et al., 2021, Supplement Section 2.3.2) on a similar LV geometry, this should be sufficient to reduce numerical errors to a minimum.

**FIGURE 1:**
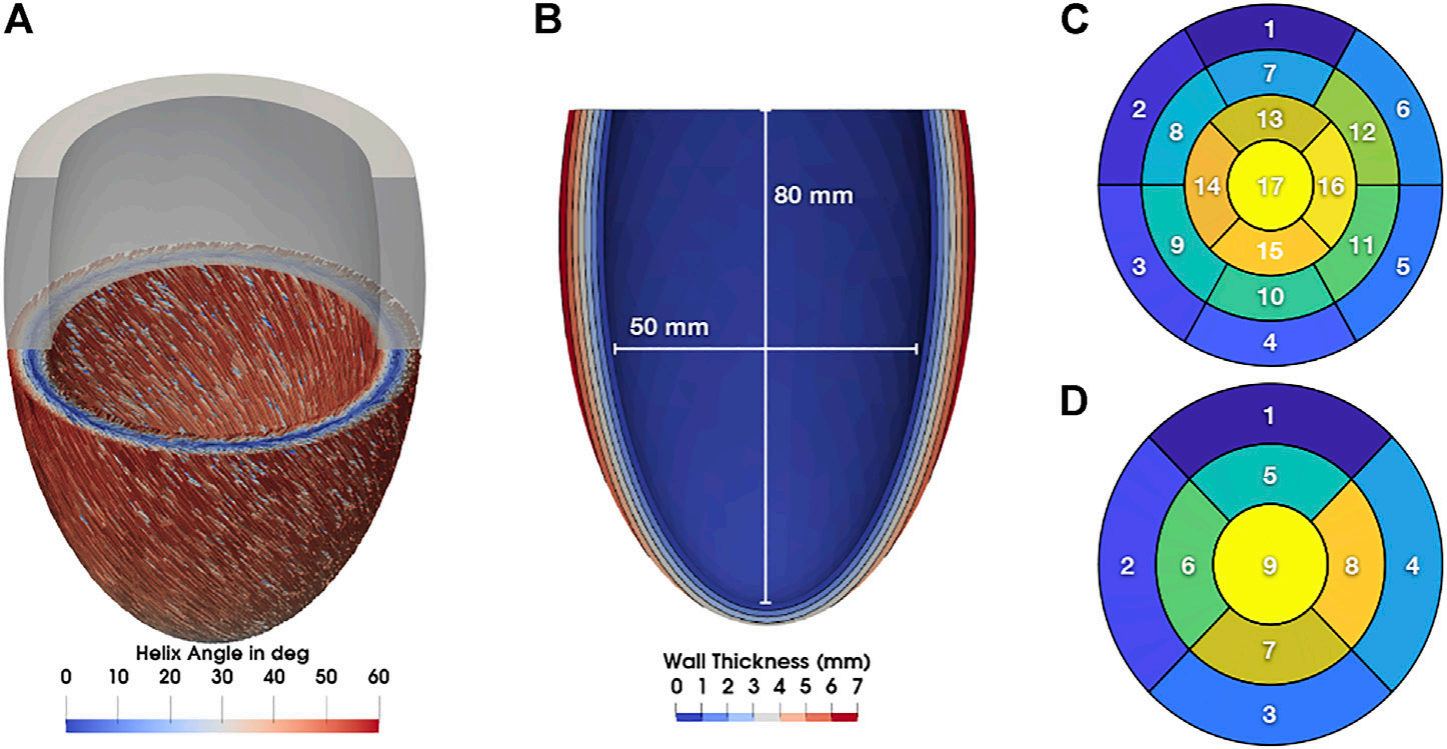
**(A)** Myocyte orientation in the LV colorized with the corresponding helix angle. **(B)** Long axis cut through the LV with contours showing the wall thickness. Additionally, lengths for the calculation of the sphericity index are shown. **(C)** 17 AHA segments. **(D)** nine NOGA XP segments.

We applied a rule-based method based on Bayer et al. (2012) ^1^ to generate the local fiber and sheet architecture **Q** = {**f**_0_, **s**_0_, **n**_0_} of the myocardium with fiber angles of 60°at the endocardium and -60°at the epicardium (Figure 1) in agreement with observations from diffusion tensor MRI of human hearts (Lombaert et al., 2012). Furthermore, we computed ventricular coordinates according to Schuler et al. (2021) and used them to separate the ventricle into the 17 segments classified by the American Heart Association (AHA; Cerqueira et al., 2002). The nine segments used by the NOGA XP system (Biosense Webster, Irvine, CA, United States) were defined equally. The NOGA XP segmentation consists of four basal segments (basoseptal, basolateral, posterobasal, anterobasal), four mid-ventricular segments (midseptal, midlateral, midposterior, midanterior), and one apical segment.

### 2.2 Electromechanical Computational Model

The kinematics of the LV are determined by solving the governing equation for the balance of linear momentum:

ρ0∂t2u−∇⋅FSu,Ta=0 inΩ0×0,T, (1)

FSu,TaN=−kepiguN onΓ0epi×0,T, (2)

FSu,TaN=−pLVtJF−TN onΓ0endo×0,T, (3)

$$\partial_{t}v\left(t\right)=C\left(t,v,z,p_{\text{LV}}\left(t\right)\right),$$ (4)

$$V_{\text{LV}}^{\text{0D}}\left(t\right)=V_{\text{LV}}^{3\text{D}}\left(t\right),$$ (5)

where we denote **F** = **I** + ∇ **u** as the deformation gradient tensor with the displacement of the myocardium **u**, *J* = det(**F**) as the Jacobian, and *ρ*_0_ as the density in the reference configuration. Furthermore, we introduce the right Cauchy-Green tensor **C** = **F** ^*T*^ **F**. The second Piola-Kirchhoff stress tensor **S** = **S** (**u**, *T*_*a*_) incorporates both, the passive and active mechanics of the myocardium using the relationship

$$S\left(u,T_{a}\right)=2\frac{\partial \Psi \left(C\right)}{\partial C}+T_{\text{a}}\left(t,\lambda_{f}\right)\frac{f_{0}⊗f_{0}}{f_{0}\cdot Cf_{0}}.$$ (6)

Passive stress in cardiac tissue is modeled using a hyperelastic strain-energy function proposed by Usyk et al. (2000):

$$\begin{array}{ll} \Psi \left(C\right) & =\frac{\kappa }{2}{\left(\log J\right)}^{2}+\frac{\mu }{2}\left(\exp\left(Q\right)-1\right),\\ Q & =b_{\text{ff}}E_{\text{ff}}^{2}+b_{\text{ss}}E_{\text{ss}}^{2}+b_{\text{nn}}E_{\text{nn}}^{2}+b_{\text{fs}}\left(E_{\text{fs}}^{2}+E_{\text{sf}}^{2}\right)+b_{\text{fn}}\left(E_{\text{fn}}^{2}+E_{\text{nf}}^{2}\right)+b_{\text{ns}}\left(E_{\text{ns}}^{2}+E_{\text{sn}}^{2}\right), \end{array}$$ (7)

with the directional components of the Green-Lagrange strain tensor $E=\frac{1}{2}\left(C-I\right)$ , bulk modulus *κ*, shear modulus *μ*, and dimensionless orthotropic scaling factors *b*_ij_, *i*, *j* ∈ *f*, *s*, *n*. Active stress due to the contraction of the cardiac muscle acts along the myocyte orientation **f**_0_ with the scalar value of the contractile force *T*_a_ = *T*_a_ (*t*, *λ*_f_). The simplified model by Niederer et al. (2011) is used to describe the temporal development of force generation:

$$T_{\text{a}}\left(t,\lambda_{f}\right)=T_{\text{peak}}\phi \left(\lambda \right)\tanh^{2}\left(\frac{t_{\text{s}}}{\tau_{c}}\right)\tanh^{2}\left(\frac{t_{\text{dur}}-t_{\text{s}}}{\tau_{r}}\right)\;for\;0<t_{\text{s}}<t_{\text{dur}},$$ (8)

$$\phi \left(\lambda_{f}\right)=\max\left\{\tanh\left(\text{ld}\left(\lambda_{f}-\lambda_{0}\right)\right),0\right\},\;\tau_{c}=\tau_{c0}+{\text{ld}}_{\text{up}}\left(1-\phi \left(\lambda_{f}\right)\right),\;t_{\text{s}}=t-t_{\text{a}}-t_{\text{emd}},$$ (9)

where *λ*_f_ is the fiber stretch, and *t*_a_ is the time of mechanical activation determined from electroanatomical mapping as detailed in Section 2.3. All parameters for the passive and active mechanics are given in Table 1.The boundary condition in Eq. (3) is imposed on the endocardium $\Gamma_{0}^{\text{endo}}$ to account for the pressure *p*_LV_(*t*) applied by the blood inside the LV. *p*_LV_(*t*) is determined by a 0D circulation model $C\left(t,v,z,p_{\text{LV}}\left(t\right)\right)$ and the coupling condition in Eq. (5) ensures volume consistency, which has to be satisfied at each time step *t* ∈ (0, *T*). Additionally, the interaction between the LV epicardium $\Gamma_{0}^{\text{epi}}$ and the surrounding tissue (Pfaller et al., 2018; Strocchi et al., 2020) is considered by the boundary condition given in Eq. (2). The contact handling algorithm proposed by Fritz et al., (2014) is used in this study. This ensures a more realistic movement of the ventricle along the long axis of the heart with improved mitral valve displacement during systole. Since the LV is under constant stress due to the flow of blood, we have to find a suitable initial stress distribution. Therefore, we first find a stress-free state of the LV by solving an inverse elasto-static problem as described in Marx et al. (2021). Then, the stress-free configuration is inflated with a pressure *p*_LV_ = 8 mmHg by solving the static problem

$$\nabla \cdot \left(F\;S\left(u,T_{a}=0\right)\right)=0\;in\;\Omega ,$$ (10)

$$F\;S\left(u,T_{a}=0\right)N=-p_{\text{LV}}\left(t\right)JF^{-T}N\;on\;\Gamma^{\text{endo}},$$ (11)

to find the displacement **u**. Finally, this displacement is used as an initial condition **u**_0_ for the problem described in Eqs 1–5.

**TABLE 1:** Input parameters for the electromechanical computational model.

Parameter	Value	Unit	Description
*Passive biomechanics*			
*ϱ*_0_	1,082	kg/m^3^	tissue density
*κ*	1	MPa	bulk modulus
*μ*	651.12	Pa	shear modulus
*b*_ff_	11	-	fiber strain scaling
*b*_ss_	4.4	-	radial strain scaling
*b*_nn_	2.2	-	cross-fiber in-plain strain scaling
*b*_fs_	7.7	-	shear strain in fiber-sheet plane scaling
*b*_fn_	6.6	-	shear strain in fiber-normal plane scaling
*b*_ns_	3.3	-	shear strain in sheet-normal plane scaling
*Active biomechanics*			
*λ*_0_	0.7	-	minimum fiber stretch
*t*_emd_	0.0	s	electromechanical delay
*T*_peak_	50	kPa	peak isometric tension
*t*_dur_	0.42	s	duration of active contraction
*τ*_*c*0_	0.14	s	base time constant of contraction
ld	5.0	-	degree of length dependence
ld_up_	0.5	s	length dependence of upstroke time
*τ*_r_	0.05	s	time constant of relaxation
*t*_cycle_	0.8	s	length of heart cycle

The mathematical model described here is a reduction of the four-chamber model presented in Gerach et al. (2021) to enable the simulation of only the LV as available from the clinical data. Nevertheless, the methods for the numerical approximation of Eqs. (1)–(5) are equivalent to the previously published four-chamber model, which is why we omit a detailed description at this point and refer the interested reader to our previous work for further details.

### 2.3 Clinical Data Integration

Jadczyk et al. (2021) performed an intra-cardiac mapping study on 30 heart failure patients with reduced ejection fraction due to an ischemic etiology. The mean age of the population was 65.4 ± 6.1 years with a higher number of male participants (*n* = 21). Mean left ventricular ejection fraction, end-diastolic volume, and end-systolic volume were 30 ± 6%, 240.5 ± 65.8 ml and 178.4 ± 49.4 ml, respectively. All individuals presented sinus rhythm and LBBB morphology on 12-lead ECG with a mean QRS duration of 168 ± 17 ms. For the enrollment of patients, the LBBB criteria of Strauss et al. (2011) including QRS duration and morphology were used. LGE-MRI showed intensities of 11.6 ± 5.2%, 6.3 ± 4.5%, and 5.4 ± 3.2% in apical, medial, and basal segments, respectively. Patients were on optimal medical therapy in accordance to the European Society of Cardiology guidelines. There was no statistically significant difference between groups. For further information, we kindly refer the reader to the original publication (Jadczyk et al., 2021).

**FIGURE 2:**
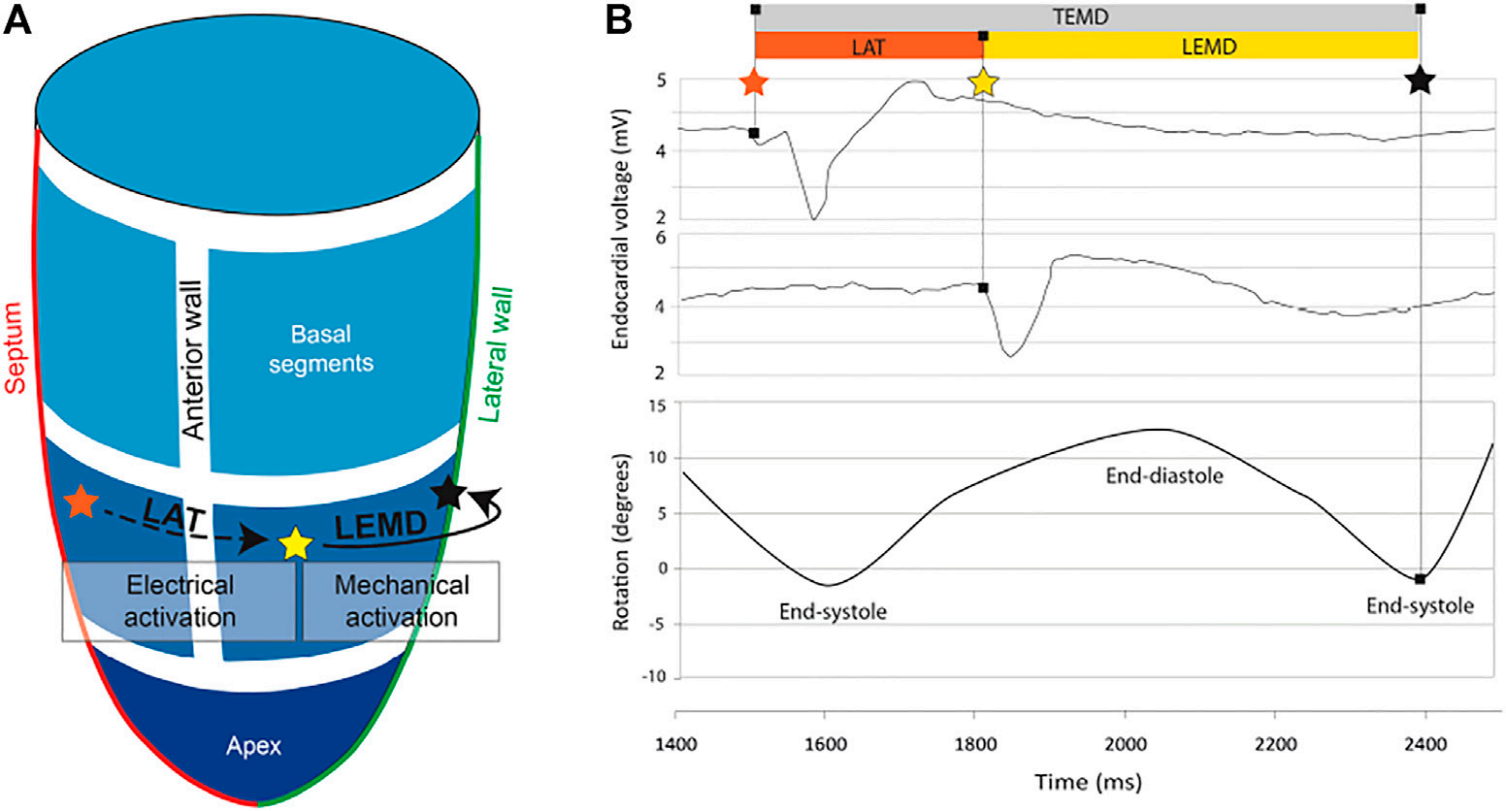
**(A)** 3D model of the left ventricle showing the relation between local activation time (LAT) and local electromechanical delay. **(B)** Electromechanical parameters of the left ventricle and how they relate to endocardial voltage and rotation. The orange star indicates the first electrical activation in the LV. The yellow star indicates measured electrical activity at another location in the LV. As the tissue at this position starts to contract, the LV begins to rotate. The time when peak rotation is reached, is symbolized by a black star.

Jadczyk et al. (2021) acquired LV end-diastolic and end-systolic volume using transthoracic echocardiography, local scar burden using LGE-MRI, and electromechanical mapping using the NOGA XP system, which allows simultaneous measurement of local electrical activity and mechanical motion. Using the catheter, local activation time (LAT) was measured as the time that passed since the first electrical activation in the LV. The time between the LAT and the measured peak systolic rotation of the point is defined as the local electromechanical delay (LEMD). Together, both of these values give the total electromechanical delay (TEMD) as shown in Figure 2.

The measurements from each patient were incorporated into the LV geometry presented in Section 2.1 by assigning the LGE-MRI data via the 17 AHA segments and LAT, LEMD, and TEMD via the nine segments of the NOGA XP system. To better differentiate between the potential influence of both, altered mechanical activation and scar burden, we first simulate all cases with only the measurements from the NOGA XP system and add local scar burden from LGE-MRI in a second run. Jadczyk et al. (2021) defined the LEMD parameter as the time interval between the local electrical activation of the segment and its peak of systolic rotation, not the onset of mechanical activation. This is an accepted approach in clinical studies of human LV mechanics (Paoletti Perini et al., 2016). Since LEMD is the only available parameter that relates to the mechanical activation including electromechanical delay, the onset of mechanical activation is set as *t*_a_ = LAT + LEMD in each segment and assigned to the center of the endocardial surface that belongs to the corresponding segment. To avoid sharp transitions between the segments due to the low resolution of the available LEMD data, all values are interpolated over the whole endocardium using Laplacian minimization (Oostendorp et al., 1989). The resulting endocardial activation is then mapped to the volumetric myocardium using nearest neighbor interpolation and propagated transmurally with a transverse conduction velocity of ${\text{CV}}_{s_{0}}=0.4$ m/s (Augustin et al., 2016). This adds an activation delay to all points **X** based on the distance from the endocardium *D*(**X**):

$$t_{\text{a}}\left(X\right)=t_{\text{a}}\left(X\in \Gamma_{0}^{\text{endo}}\right)+\frac{D\left(X\right)}{{\text{CV}}_{s_{0}}},$$ (12)

resulting in a maximal transmural delay of epicardial activation of 17.5 ms at the base where the wall thickness is 7 mm.

Local scar burden was incorporated by the percentage of enhanced area determined from LGE-MRI measurements on a segment-by-segment basis. It is assumed that the myocardium in enhanced areas was subject to remodeling processes and thus shows a decreased contractility. To reflect this in our model, the maximal contractility *T*_peak_ was reduced by the same amount as the percentage of the measured LGE intensity in the respective segment.

### 2.4 Evaluation Metrics

The main focus of this study is to determine different rotation patterns in the LV emerging from locally altered electromechanical delay. Therefore, we need to calculate the rotation angle as well as rotation direction of points located on the endocardial wall with respect to the heart’s long axis. Notice that only endocardial points are evaluated since the clinical data was measured with an intracardiac mapping device. In our idealized LV model, the long axis corresponds to the *z*-axis of the global coordinate system in basal-apical direction. Hence, we extract all endocardial points $r=X\in \Gamma_{0}^{\text{endo}}$ in each segment, project them onto the (*x*, *y*) plane, and use the relationship

$$\cos\alpha =\frac{r_{\text{ED}}\cdot r_{\text{ES}}}{\left|r_{\text{ED}}|\cdot |r_{\text{ES}}\right|}$$ (13)

to calculate the angular displacement *α* from end-diastole (ED) to end-systole (ES). The direction of rotation was determined through

$$\det\left(r_{\text{ED}},r_{\text{ES}}\right)$$ (14)

with values >0 indicating counterclockwise rotation and values <0 indicating clockwise rotation. ED and ES states are determined from the pressure-volume relationship *p*_LV_(*V*_LV_) resulting from the 0D circulation model C . Additionally, we use Lagrangian particle tracking to visualize three dimensional trajectories of points located on the endocardium. Finally, the simulations are classified into one of two categories based on their rotational behavior: 1) wringing rotation denoted as Group A when basal segments show clockwise rotation and apical segments show counterclockwise rotation; 2) rigid-body-type (RBT) rotation denoted as Group B when the segments show either predominantly clockwise or predominantly counterclockwise rotation. The latter is realized by using a threshold of ±3° to decide whether a segment is rotating clockwise or counterclockwise, respectively. If nine or more segments show the same rotation pattern, the case is assigned to Group B.

## 3 Results

We studied how locally altered electromechanical activation determines the diverse LV rotation patterns observed in HFrEF patients diagnosed with LBBB using in-silico models of the LV in a total of 31 cases. 30 of them were informed by patient specific measurements of LAT and LEMD using the measured data reported in Jadczyk et al. (2021) with the NOGA XP system as well as scar burden using the percentage of enhanced area from LGE-MRI. Additionally, we simulated a Control case without variations in LEMD and without scar tissue. For three of the clinical cases, simulations failed with the parameter set given in Table 1. Thus, simulation results are reported for 27 clinical cases. Since the rotational analysis of the simulations with and without local scar burden showed only minor differences and the classification was the same, results are reported for the cases including local scar burden unless otherwise stated.

**FIGURE 3:**
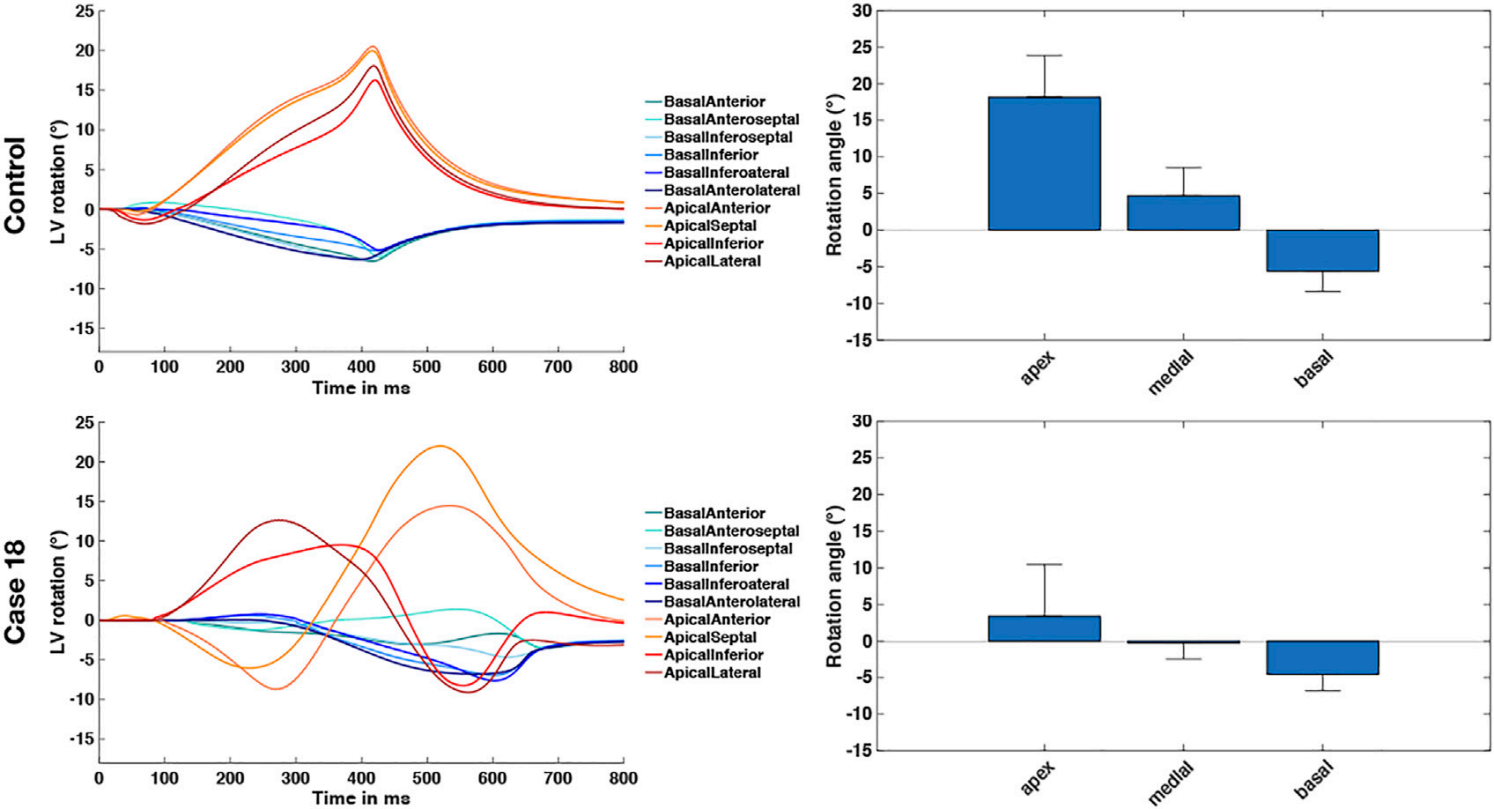
Rotational dynamics of the basal and apical segments during one whole heart beat (left panel) and sectorial mean end-systolic rotation angle (right panel). The Control case is shown on the top and represents wringing rotation. Case 18 is shown on the bottom and was classified as RBT rotation.

Based on Eqs 13, 14, we calculated the angle of rotation for all endocardial points and evaluated sectorial (basal, medial, apical) as well as segmental (17 AHA segments) mean values in each time step. Figure 3 shows the dynamic rotational behavior of the LV in the basal and apical segments (left panel) as well as the sectorial mean angle of rotation during end-systole (right panel).

**FIGURE 4:**
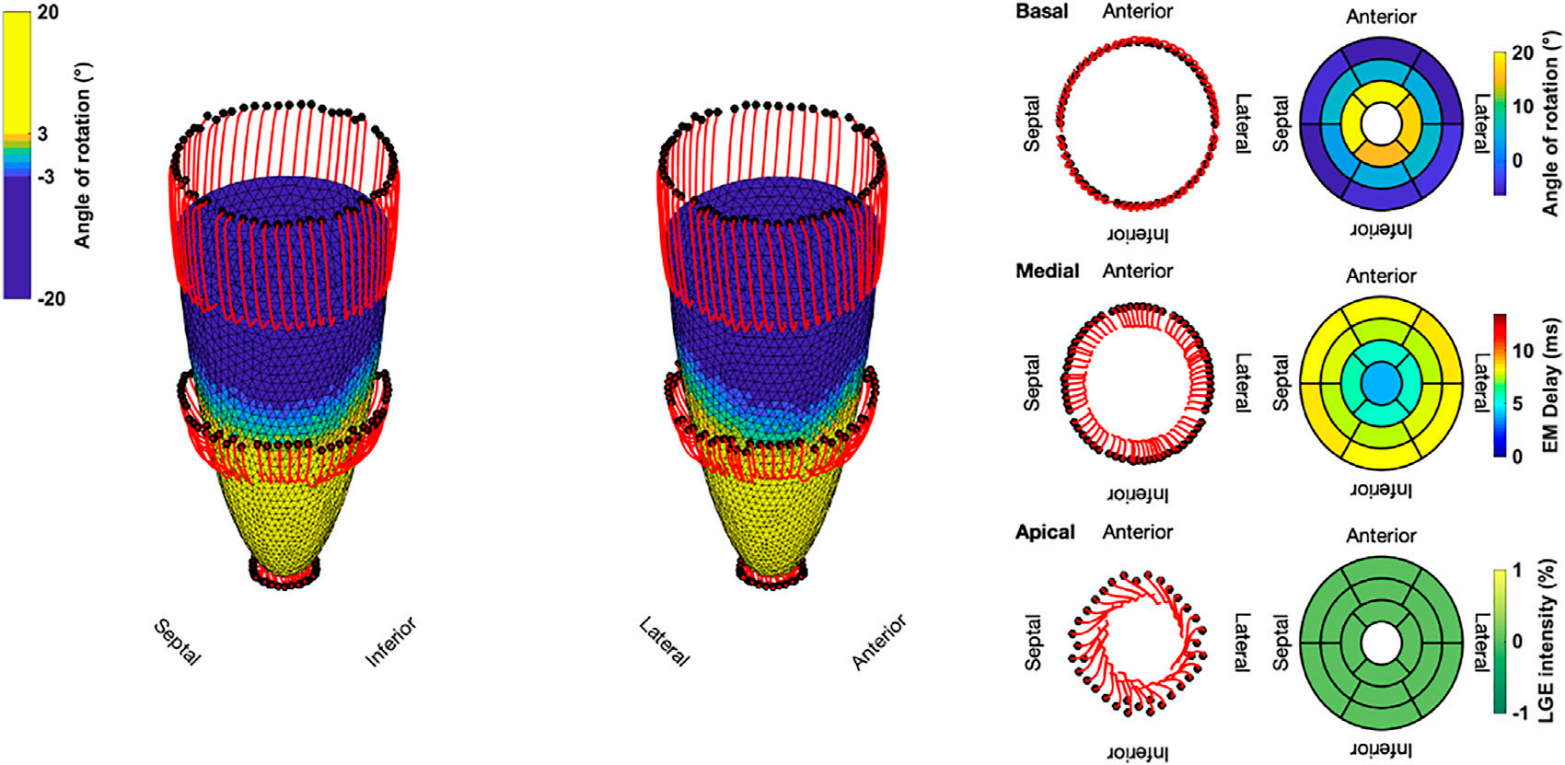
Three-dimensional trajectories (red lines) of selected points on the endocardium in the basal, medial, and apical sectors for the Control case. The endocardial surface during end-systole is shown with rotational values depicted on the faces of the mesh. Solid black dots represent the initial positions. Bullseye plots show the segmential mean values of the angle of rotation, electromechanical (EM) delay, and LGE intensity.

The results for the Control case are shown at the top. In the first 100 ms, the LV experienced a short untwist meaning that apical segments rotated clockwise with up to −3° and basal segments rotated counterclockwise with up to 1°. Right after this first phase, apical and basal segments start to rotate in the opposite direction until end-systole is reached (at about 410 ms). The mean angle of rotation was 18°, 5°, and −6° for the apical, medial, and basal sectors, respectively. At the bottom of Figure 3, the results for Case 18 are shown. Here, we could not observe a clear phase of untwisting. However, apical segments show opposite directions of rotation throughout the simulated heartbeat. First, anterior and septal segments showed up to −10° of clockwise rotation while lateral and inferior segments rotated counterclockwise with up to 12°. Towards end-systole however, the direction of rotation switches to the exact opposite behavior, leading to a predominantly clockwise rotation of basal and apical segments. This is reflected in the sectorial mean angle of end-systolic rotation as well. The apical sector showed significantly lower rotation compared to the Control case, whereas the medial sector switched from counterclockwise to clockwise rotation. Basal segments showed slightly lower angles of rotation compared to Control. In the Lagrangian particle tracking of the Control case (Figure 4), the wringing rotation could be clearly observed. Apical segments distinctly showed counterclockwise rotation up until end-systole. Additionally, we observed a translational movement towards the lateral side of the LV. In the medial and basal segments, the contraction was much more symmetrical and the myocardium in these segments rotated predominantly in a clockwise manner. However, the rotation is not as dominant as the shortening in the long axis in these segments.

**FIGURE 5:**
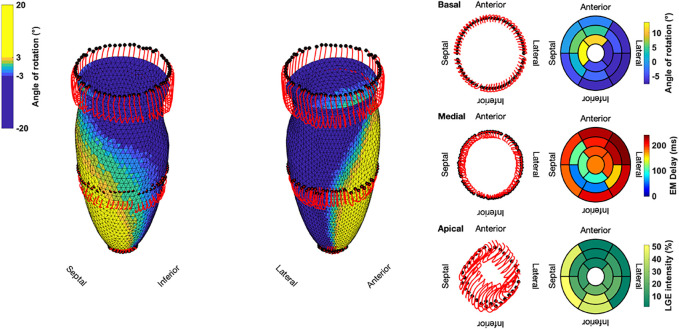
Three-dimensional trajectories (red lines) of selected points on the endocardium in the basal, medial, and apical sectors for Case 18. The endocardial surface during end-systole is shown with rotational values depicted on the faces of the mesh. Solid black dots represent the initial positions. Bullseye plots show the segmential mean values of the angle of rotation, electromechanical (EM) delay, and LGE intensity.

Compared to the Control case, Case 18 showed a markedly different contraction pattern (Figure 5). Most notably, the symmetry of the homogeneous contraction pattern is lost. Inferior-lateral segments in the basal and medial sectors displayed less wall thickening, yet more shortening in the long axis compared to the Control case. Apical segments underwent a significant translation towards the anterior-lateral side of the LV with a more pronounced clockwise rotation. Due to the higher LGE intensities in basal septal and basal inferior segments, the LV shows less wall thickening and less longitudinal shortening compared to the Control case.

**FIGURE 6:**
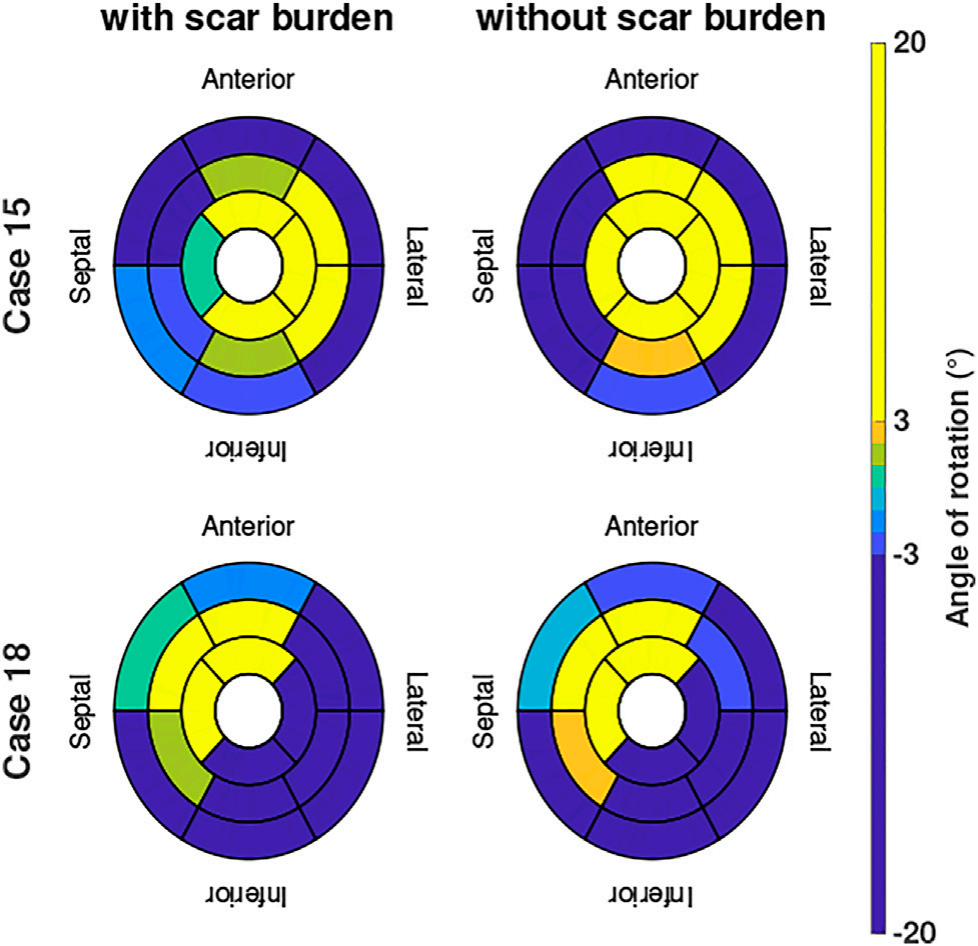
Angle of rotation for Cases 15 and 18 shown in bullseye perspective for simulations with and without local scar burden.

Two examples of the difference in the evaluated angle of rotation are given in Figure 6 in case we include or exclude local scar burden in the simulations. Case 15 (patient with the overall highest intensity values in the LGE-MRI data) showed slightly smaller rotation angles in some segments when local scar burden is incorporated into the model. With up to 5°, the change in the angle of rotation is largest in apical and medial segments. However, no change in the direction of rotation was observed. For Case 18, we observed similar changes in the magnitude of peak rotation. Angular differences occured mostly in segments with increased LGE intensity.

**FIGURE 7:**
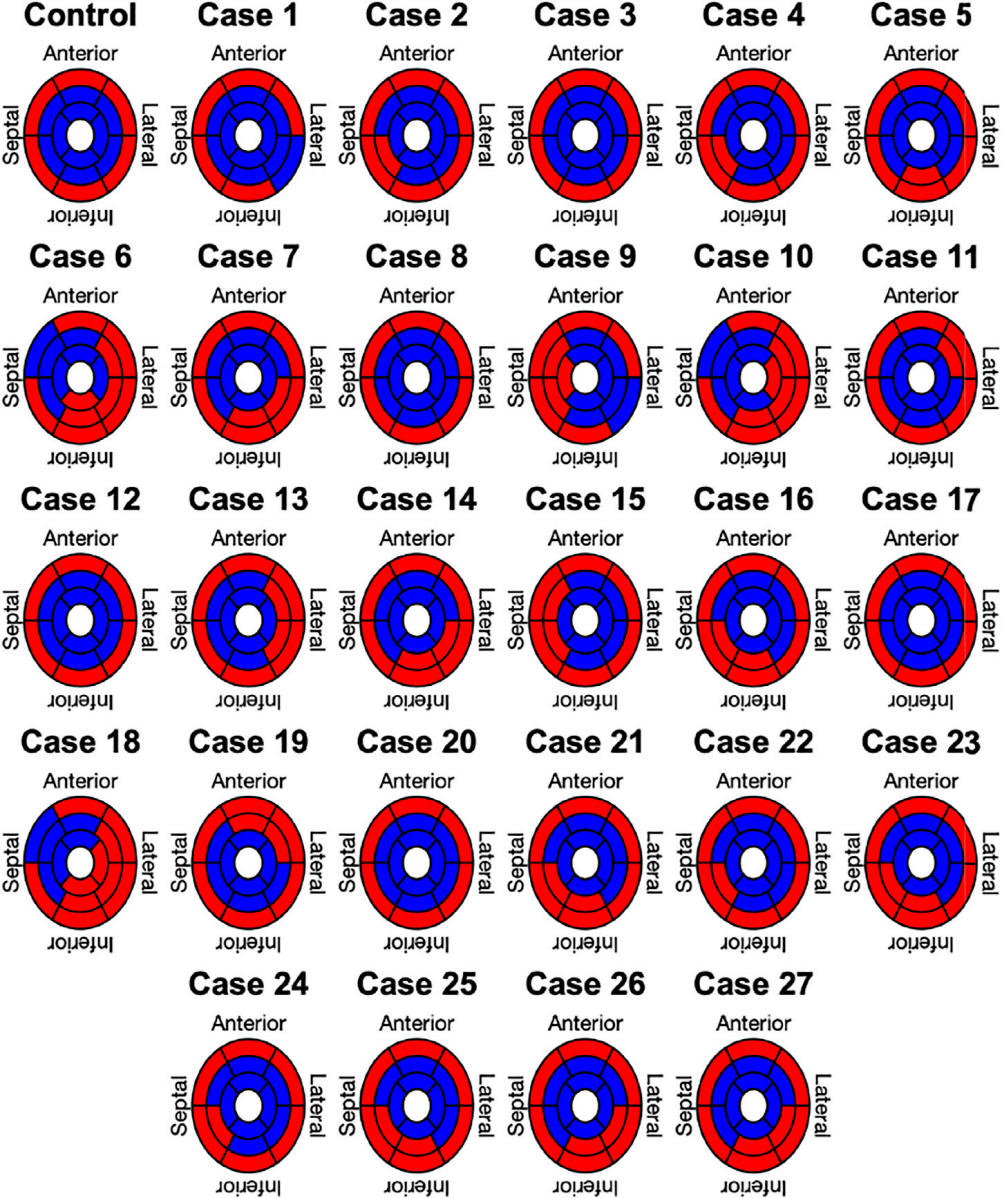
Segment based analysis of end-systolic rotation around the long axis of the LV. A binary classification was used in each segment to determine clockwise (red) or counterclockwise (blue) rotation.

Lastly, the results of the binary classification for each of the 17 AHA segments in the Control case and the 27 clinically informed simulations are shown in Figure 7. Each segment was classified by its mean angle of rotation during end-systole. The color red depicts clockwise rotation and blue counterclockwise rotation. Cases with the majority of segments (≥9) rotating clockwise were classified as RBT rotation (Group B), which was the case for three cases (6, 10, 18). No case was classified in Group B with a predominantly counterclockwise rotation, since the basal segments rotated clockwise in the majority of cases. One repeating pattern in those three cases is that the lateral-inferior side of the LV was predominantly rotating in clockwise direction, while the anterior-septal side was dominated by counterclockwise rotation. All other cases were classified as wringing rotation (Group A). Based on the clinical recordings directly, Jadczyk et al. (2021) classified 20% of the patients in Group A (*n* = 6) and 80% in Group B (*n* = 24). Furthermore, 73% (*n* = 22) of patients in Group B showed clockwise RBT rotation and predominantly counterlockwise RBT rotation was observed in 7% (*n* = 2). Compared to the clinical classification, the in silico model results yielded matching classifications for Group A in 50% (*n* = 3) of the cases and in 0% of the cases for Group B.

## 4 Discussion

We presented results of a total of 27 mechanical simulations of an idealized LV that were informed by electroanatomical maps recorded with the NOGA XP system and additional LGE-MRI data. Results of one additional simulation without a patient specific input served as a control case. We evaluated the angle of rotation of each point on the endocardium and accumulated mean values on a segmental (17 AHA segments) and sectorial (basal, medial, apical) basis. Based on the segmental mean values, we determined the direction of rotation to be either clockwise (negative rotation values) or counterclockwise (positive rotation values). Finally, each simulation was classified as wringing type rotation (Group A) or RBT rotation (Group B) and compared to the clinically assessed classification.

The Control case was parameterized to yield a physiological contraction pattern that could be used as a comparison since all other cases involve data from patients suffering from HFrEF and LBBB. Hence, we presumed the electrical activation to be synchronized such that the subendocardial mechanical contraction is homogeneous throughout the LV and spreading transmurally towards the epicardium. The evaluated rotation pattern concurs with observations made in healthy individuals quantitatively and qualitatively (Stöhr et al., 2016; Lehmonen et al., 2020). First, we observed a brief untwisting (clockwise rotation of the apex and counterclockwise rotation of the base) of the LV during isovolumetric contraction. This has been observed in clinical measurements as well (Sengupta et al., 2008; Omar et al., 2015) and is linked to the initial contraction of the subendocardial layer followed by the contraction of the subepicardial layer. During ejection, the LV starts to twist normally (counterclockwise rotation of the apex and clockwise rotation of the base). Kocabay et al. (2014) reported mean rotational values of −6.9° for the base and 13° for the apex in 247 healthy volunteers using two-dimensional speckle-tracking echocardiography. With up to −6°, the Control case in this study matches these data well at the base. However, the up to 20° of rotation at the apex is larger. This can be explained by numerous factors, e.g. it is known that preload, afterload, contractility and age have an influence on the twist angle (Sengupta et al., 2008; Omar et al., 2015). Another significant factor is the choice of the apical imaging plane, since rotation values can vary widely depending on where the rotation is measured (van Dalen et al., 2008b; Stöhr et al., 2016). Based on these facts, we think that the Control case successfully represents physiological contraction in humans.

Electrical activation and consequently mechanical contraction of the LV in the pathological cases was determined by patient specific measurements of electroanatomical maps. LAT and LEMD were directly integrated into the LV model using the segments defined by the NOGA XP catheter system. LGE-MRI intensity was used as a surrogate for scar tissue in the LV. Typically, scar tissue undergoes a remodeling process that involves build-up of collagen in the myocardium (Jugdutt et al., 1996), which can result in a reorganization of the underlying fiber structure. Thus, an acknowledged way of modeling scar tissue is to impose an isotropic fiber structure with increased stiffness in the constitutive model (Niederer et al., 2011). Since we only had access to the LGE intensity as a percentage in each of the 17 AHA segments and we used the same geometrical model for all cases, it was not possible to easily implement it this way. Instead, we decided to reduce the active tension in the respective segment by the same percentage as the LGE intensity was increased with respect to the mean blood pool value (image intensity ratio, IIR). Thus, for IIR = 1.2 (20% above the mean blood pool intensity), the active tension was reduced by 20% to 0.8 × the reference value. This approach is motivated by the fact that in case of unchanged contractility, a stiffer myocardium would result in less deformation. However, this is a simplification due to the nonlinear nature of the constitutive law given in Eq. 7. Furthermore, we effectively smooth the effect of local scar tissue over the entire segment. Other than the altered electromechanical delay captured by the LEMD values from electroanatomical mapping and the aforementioned LGE intensity to capture the effect of scar tissue, no further patho-mechanisms were considered in the simulations. Hence, the emerging rotation patterns in the pathological cases originate from these changes only.

The classification into Group A (wringing rotation) and Group B (RBT rotation) was based on the evaluation of the mean angle of rotation in the 17 AHA segments. It clearly showed that most cases were classified into Group A. With the exception of 3 cases, all cases were classified different than in the classification based directly on the clinical rotation data, which means we cannot confirm the hypothesis postulated in Jadczyk et al. (2021) with the in silico model presented in this study. However, we identified 3 cases with predominantly clockwise rotation. Additionally to the basal segments, the medial and apical inferior-lateral side of the LV started to rotate clockwise instead of counterclockwise. Noticeably, this was accompanied by a significant translational movement of the apical region towards the anterior-lateral side of the LV. This can potentially skew the results when Eqs 13, 14 are used to determine the angle and direction of rotation around a fixed axis (Carreras et al., 2011), since a translation can be mistaken as a rotation. Nonetheless, the rotational dynamics shown in Figure 3 of Case 18 show a similar pattern compared to a HFrEF case in Omar et al. (2015). There, apical rotation is reduced and undergoes both, a counterclockwise and then clockwise rotation.

Furthermore, the following limitations apply to our study:

1. LEMD was defined as the time interval between the local electrical activation of the segment and its peak systolic rotation, not the onset of mechanical activation. If we assume a similar length of active contraction in each segment, this should not pose a problem and LEMD can be interpreted as a local delay. However, different contractility can be expected especially in segments that show enhanced regions in LGE-MRI due to scar tissue and the associated tissue remodeling. This is especially important, since we observe a significantly longer time interval between electrical activation and peak systolic rotation in our simulations (489 ± 45 ms compared to the 369 ± 59 ms in the clinical measurements).
2. The presented in silico model neglects the influence of the atria and the right ventricle on rotation of the LV. Atrial data was not available for these patients at all and for the right ventricle no electroanatomical mapping data was available. Therefore, we decided to use the same LV geometrical model for all patients to isolate the effect of electromechanical activation on rotation. Especially the absence of the right ventricle might be crucial to why we can not observe clear rigid body rotation in the in silico model. The patients in the clinical cohort all presented with LBBB, meaning that the right ventricle contracted first. This might result in a lateral displacement of the septum. Consequently, an initial fraction of the active force generated by the LV contraction is spent on reversing this displacement instead of storing the entire energy in torque which ultimately results in less rotation. In our model this is not considered, which is why we might not be able to observe rigid body rotation.
3. A reason for the small differences in simulations in which scar burden was considered could be the simple way of transferring LGE intensity directly into a decrease in contractility. Although it is a straightforward approach and easy to implement, scar tissue is more complex and typically accompanied by other pathomechanisms in addition to a reduction in contractility. For example, fiber orientation in the LV of patients with dilated cardiomyopathy is typically reorganized Eggen et al. (2009). Furthermore, the stiffness of the myocardium increases and electrical conductivity decreases due to extracellular matrix remodeling Bollen et al. (2017). A detailed investigation of the effect of these additional mechanisms requires personalized LV anatomical models including information on pathological changes and was beyond the scope of this study, which focuses on the effect of dyssynchronous activation.

In conclusion, we combined a state-of-the-art model of heart mechanics and *in vivo* data of 30 patients to analyze rotational dynamics in the LV. The main aim of this study was to investigate the specific hypothesis that dyssynchrony alone affects the kinetics of the LV in patients with HFrEF and LBBB in a way that rotational behavior is qualitatively changed. If this was the case, we would have been able to reproduce the mechanical behavior observed in the NOGA XP cardiac mapping system *in vivo*. The fact that this hypothesis was falsified shows that it is not the electromechanical activation sequence alone that determines rotational behavior suggesting that additional mechanisms are involved. Implications are that further research is required to fully understand the determinants of rotational behavior and that these further (and currently unknown) mechanisms are likely also important to be addressed therapeutically.

## Data Availability

The original contributions presented in the study are included in the article/Supplementary Material, further inquiries can be directed to the corresponding author.

## Footnotes

1 This implementation of the algorithm was used: https://doi.org/10.5281/zenodo.4606575

## References

[B1] Augustin C. M. Neic A. Liebmann M. Prassl A. J. Niederer S. A. Haase G. (2016). Anatomically Accurate High Resolution Modeling of Human Whole Heart Electromechanics: A Strongly Scalable Algebraic Multigrid Solver Method for Nonlinear Deformation. J. Comput. Phys. 305, 622–646. 10.1016/j.jcp.2015.10.045 26819483 PMC4724941

[B2] Bayer J. D. Blake R. C. Plank G. Trayanova N. A. (2012). A Novel Rule-Based Algorithm for Assigning Myocardial Fiber Orientation to Computational Heart Models. Ann. Biomed. Eng. 40, 2243–2254. 10.1007/s10439-012-0593-5 22648575 PMC3518842

[B3] Bollen I. A. E. Ehler E. Fleischanderl K. Bouwman F. Kempers L. Ricke-Hoch M. (2017). Myofilament Remodeling and Function Is More Impaired in Peripartum Cardiomyopathy Compared with Dilated Cardiomyopathy and Ischemic Heart Disease. Am. J. Pathol. 187, 2645–2658. 10.1016/j.ajpath.2017.08.022 28935576

[B4] Carreras F. Garcia-Barnes J. Gil D. Pujadas S. Li C. H. Suarez-Arias R. (2011). Left Ventricular Torsion and Longitudinal Shortening: Two Fundamental Components of Myocardial Mechanics Assessed by Tagged Cine-MRI in normal Subjects. Int. J. Cardiovasc. Imaging 28, 273–284. 10.1007/s10554-011-9813-6 21305357

[B5] Cerqueira M. D. Weissman N. J. Dilsizian V. Jacobs A. K. Kaul S. Laskey W. K. (2002). Standardized Myocardial Segmentation and Nomenclature for Tomographic Imaging of the Heart. A Statement for Healthcare Professionals from the Cardiac Imaging Committee of the Council on Clinical Cardiology of the American Heart Association. J. Nucl. Cardiol. 9, 240–245. 10.1067/mnc.2002.123122 11986572

[B6] Daubert J.-C. Daubert J.-C. Saxon L. Adamson P. B. Auricchio A. Berger R. D. (2012). 2012 EHRA/HRS Expert Consensus Statement on Cardiac Resynchronization Therapy in Heart Failure: Implant and Follow-Up Recommendations and Management: A Registered branch of the European Society of Cardiology (ESC), and the Heart Rhythm Society; and in Collaboration with the Heart Failure Society of America (HFSA), the American Society of Echocardiography (ASE), the American Heart Association (AHA), the European Association of Echocardiography (EAE) of the ESC and the Heart Failure Association of the ESC (HFA). * Endorsed by the Governing Bodies of AHA, ASE, EAE, HFSA, HFA, EHRA, and HRS. Europace 14, 1236–1286. 10.1093/europace/eus222 22930717

[B7] Eggen M. D. Swingen C. M. Iaizzo P. A. (2009). “Analysis of Fiber Orientation in normal and Failing Human Hearts Using Diffusion Tensor Mri,” in 2009 IEEE international symposium on biomedical imaging: from nano to macro (IEEE), 642–645. 10.1109/isbi.2009.5193129

[B8] Fritz T. Wieners C. Seemann G. Steen H. Dössel O. (2014). Simulation of the Contraction of the Ventricles in a Human Heart Model Including Atria and Pericardium. Biomech. Model. Mechanobiol 13, 627–641. 10.1007/s10237-013-0523-y 23990017

[B9] Gerach T. Schuler S. Fröhlich J. Lindner L. Kovacheva E. Moss R. (2021). Electro-mechanical Whole-Heart Digital Twins: A Fully Coupled Multi-Physics Approach. Mathematics 9, 1247. 10.3390/math9111247

[B10] Geuzaine C. Remacle J.-F. (2009). Gmsh: A 3-d Finite Element Mesh Generator with Built-In Pre- and post-processing Facilities. Int. J. Numer. Meth. Engng. 79, 1309–1331. 10.1002/nme.2579

[B11] Jadczyk T. Kurzelowski R. Golba K. S. Wilczek J. Caluori G. Maffessanti F. (2021). Local Electromechanical Alterations Determine the Left Ventricle Rotational Dynamics in CRT-Eligible Heart Failure Patients. Sci. Rep. 11. 10.1038/s41598-021-82793-1 PMC7865069 33547401

[B12] Jugdutt B. I. Joljart M. J. Khan M. I. (1996). Rate of Collagen Deposition during Healing and Ventricular Remodeling after Myocardial Infarction in Rat and Dog Models. Circulation 94, 94–101. 10.1161/01.cir.94.1.94 8964124

[B13] Kocabay G. Muraru D. Peluso D. Cucchini U. Mihaila S. Padayattil-Jose S. (2014). Normal Left Ventricular Mechanics by Two-Dimensional Speckle-Tracking Echocardiography. Reference Values in Healthy Adults. Revista Española de Cardiología (English Edition) 67, 651–658. 10.1016/j.rec.2013.12.009 25037544

[B14] Leclercq C. Burri H. Curnis A. Delnoy P. P. Rinaldi C. A. Sperzel J. (2019). Cardiac Resynchronization Therapy Non-responder to Responder Conversion Rate in the More Response to Cardiac Resynchronization Therapy with Multipoint Pacing (MORE-CRT MPP) Study: Results from Phase I. Eur. Heart J. 40, 2979–2987. 10.1093/eurheartj/ehz109 30859220

[B15] Lehmonen L. Jalanko M. Tarkiainen M. Kaasalainen T. Kuusisto J. Lauerma K. (2020). Rotation and Torsion of the Left Ventricle with Cardiovascular Magnetic Resonance Tagging: Comparison of Two Analysis Methods. BMC Med. Imaging 20. 10.1186/s12880-020-00473-4 PMC7329530 32611329

[B16] Lombaert H. Peyrat J. Croisille P. Rapacchi S. Fanton L. Cheriet F. (2012). Human Atlas of the Cardiac Fiber Architecture: Study on a Healthy Population. IEEE Trans. Med. Imaging 31, 1436–1447. 10.1109/TMI.2012.2192743 22481815

[B17] Marx L. Niestrawska J. A. Gsell M. A. F. Caforio F. Plank G. Augustin C. M. (2021). Efficient Identification of Myocardial Material Parameters and the Stress-free Reference Configuration for Patient-specific Human Heart Models. Quantitative Biol. 10.48550/arXiv.2101.04411

[B18] Niederer S. A. Plank G. Chinchapatnam P. Ginks M. Lamata P. Rhode K. S. (2011). Length-dependent Tension in the Failing Heart and the Efficacy of Cardiac Resynchronization Therapy. Cardiovasc. Res. 89, 336–343. 10.1093/cvr/cvq318 20952413

[B19] Omar A. M. S. Vallabhajosyula S. Sengupta P. P. (2015). Left Ventricular Twist and Torsion. Circ. Cardiovasc. Imaging 8. 10.1161/CIRCIMAGING.115.003029 25991575

[B20] Oostendorp T. F. van Oosterom A. Huiskamp G. (1989). Interpolation on a Triangulated 3d Surface. J. Comput. Phys. 80, 331–343. 10.1016/0021-9991(89)90103-4

[B21] Paoletti Perini A. Sacchi S. Votta C. D. Lilli A. Attanà P. Pieragnoli P. (2016). Left Ventricular Rotational Dyssynchrony before Cardiac Resynchronization Therapy. J. Cardiovasc. Med. 17, 469–477. 10.2459/jcm.0000000000000391 27116377

[B22] Pfaller M. R. Hörmann J. M. Weigl M. Nagler A. Chabiniok R. Bertoglio C. (2018). The Importance of the Pericardium for Cardiac Biomechanics: from Physiology to Computational Modeling. Biomech. Model. Mechanobiol 18, 503–529. 10.1007/s10237-018-1098-4 30535650

[B23] Popescu B. A. Beladan C. C. Călin A. Muraru D. Deleanu D. Roşca M. (2009). Left Ventricular Remodelling and Torsional Dynamics in Dilated Cardiomyopathy: Reversed Apical Rotation as a Marker of Disease Severity. Eur. J. Heart Fail. 11, 945–951. 10.1093/eurjhf/hfp124 19789397

[B24] Rüssel I. K. Götte M. J. W. Bronzwaer J. G. Knaapen P. Paulus W. J. van Rossum A. C. (2009b). Left Ventricular Torsion. JACC: Cardiovasc. Imaging 2, 648–655. 10.1016/j.jcmg.2009.03.001 19442954

[B25] Rüssel I. K. Götte M. J. W. de Roest G. J. Marcus J. T. Tecelão S. R. Allaart C. P. (2009a). Loss of Opposite Left Ventricular Basal and Apical Rotation Predicts Acute Response to Cardiac Resynchronization Therapy and Is Associated with Long-Term Reversed Remodeling. J. Card. Fail. 15, 717–725. 10.1016/j.cardfail.2009.04.007 19786261

[B26] Rüssel I. K. Götte M. J. W. (2011). New Insights in LV Torsion for the Selection of Cardiac Resynchronisation Therapy Candidates. Neth. Heart J. 19, 386–391. 10.1007/s12471-011-0136-y 21562790 PMC3167247

[B27] Sade L. E. Demir Ö. Atar I. Müderrisoglu H. Özin B. (2008). Effect of Mechanical Dyssynchrony and Cardiac Resynchronization Therapy on Left Ventricular Rotational Mechanics. Am. J. Cardiol. 101, 1163–1169. 10.1016/j.amjcard.2007.11.069 18394452

[B28] Schuler S. Pilia N. Potyagaylo D. Loewe A. (2021). Cobiveco: Consistent Biventricular Coordinates for Precise and Intuitive Description of Position in the Heart - with MATLAB Implementation. Med. Image Anal. 74, 102247. 10.1016/j.media.2021.102247 34592711

[B29] Sengupta P. P. Krishnamoorthy V. K. Korinek J. Narula J. Vannan M. A. Lester S. J. (2007). Left Ventricular Form and Function Revisited: Applied Translational Science to Cardiovascular Ultrasound Imaging. J. Am. Soc. Echocardiography 20, 539–551. 10.1016/j.echo.2006.10.013 PMC1951787 17485001

[B30] Sengupta P. P. Tajik A. J. Chandrasekaran K. Khandheria B. K. (2008). Twist Mechanics of the Left Ventricle. JACC: Cardiovasc. Imaging 1, 366–376. 10.1016/j.jcmg.2008.02.006 19356451

[B31] Setser R. M. Kasper J. M. Lieber M. L. Starling R. C. McCarthy P. M. White R. D. (2003). Persistent Abnormal Left Ventricular Systolic Torsion in Dilated Cardiomyopathy after Partial Left Ventriculectomy. J. Thorac. Cardiovasc. Surg. 126, 48–55. 10.1016/S0022-5223(03)00050-3 12878938

[B32] Sillanmäki S. Lipponen J. A. Tarvainen M. P. Laitinen T. Hedman M. Hedman A. (2018). Relationships between Electrical and Mechanical Dyssynchrony in Patients with Left Bundle branch Block and Healthy Controls. J. Nucl. Cardiol. 26, 1228–1239. 10.1007/s12350-018-1204-0 29423906

[B33] Stöhr E. J. Shave R. E. Baggish A. L. Weiner R. B. (2016). Left Ventricular Twist Mechanics in the Context of normal Physiology and Cardiovascular Disease: a Review of Studies Using Speckle Tracking Echocardiography. Am. J. Physiology-Heart Circulatory Physiol. 311, H633–H644. 10.1152/ajpheart.00104.2016 27402663

[B34] Strauss D. G. Selvester R. H. Wagner G. S. (2011). Defining Left Bundle Branch Block in the Era of Cardiac Resynchronization Therapy. Am. J. Cardiol. 107, 927–934. 10.1016/j.amjcard.2010.11.010 21376930

[B35] Strocchi M. Gsell M. A. F. Augustin C. M. Razeghi O. Roney C. H. Prassl A. J. (2020). Simulating Ventricular Systolic Motion in a Four-Chamber Heart Model with Spatially Varying Robin Boundary Conditions to Model the Effect of the Pericardium. J. Biomech. 101, 109645. 10.1016/j.jbiomech.2020.109645 32014305 PMC7677892

[B36] Usyk T. P. Mazhari R. McCulloch A. D. (2000). Effect of Laminar Orthotropic Myofiber Architecture on Regional Stress and Strain in the Canine Left Ventricle. J. Elasticity 61, 143–164. 10.1023/A:1010883920374

[B37] van Dalen B. M. Caliskan K. Soliman O. I. I. Nemes A. Vletter W. B. Ten Cate F. J. (2008a). Left Ventricular Solid Body Rotation in Non-compaction Cardiomyopathy: a Potential New Objective and Quantitative Functional Diagnostic Criterion? Eur. J. Heart Fail. 10, 1088–1093. 10.1016/j.ejheart.2008.08.006 18815069

[B38] van Dalen B. M. Vletter W. B. Soliman O. I. I. ten Cate F. J. Geleijnse M. L. (2008b). Importance of Transducer Position in the Assessment of Apical Rotation by Speckle Tracking Echocardiography. J. Am. Soc. Echocardiography 21, 895–898. 10.1016/j.echo.2008.02.001 18356018

